# Draft Genome Sequence of the Oxytetracycline-Producing Bacterium *Streptomyces rimosus* ATCC 10970

**DOI:** 10.1128/genomeA.00063-13

**Published:** 2013-03-07

**Authors:** Florence E. Pethick, Alison C. MacFadyen, Zhenyu Tang, Vartul Sangal, Tze-Tze Liu, Ju Chu, Gregor Kosec, Hrvoje Petkovic, Meijin Guo, Ralph Kirby, Paul A. Hoskisson, Paul R. Herron, Iain S. Hunter

**Affiliations:** a Strathclyde Institute of Pharmacy and Biomedical Sciences, University of Strathclyde, Glasgow, United Kingdom; b State Key Laboratory of Bioreactor Engineering, East China University of Science and Technology, Shanghai, China; c Genome Research Center, National Yang-Ming University, Taipei, Taiwan; d Acies Bio, Ltd., Ljubljana, Slovenia; e Department of Life Sciences, Institute of Genome Science, National Yang-Ming University, Taipei, Taiwan

## Abstract

We report the draft genome of *Streptomyces rimosus* (ATCC 10970), a soil isolate that produces oxytetracycline, a commercially important and clinically useful antibiotic.

## GENOME ANNOUNCEMENT

*Streptomyces rimosus* is a Gram-positive, aerobic, filamentous actinobacterium. It was reported in 1950 and was patented (1) as the founder strain for the production of the antibiotic oxytetracycline (OTC) (2). Recombination was recognized (3) and a rudimentary genetic map was deduced (4). The pharmaceutical industry set out to improve the commercial productivity of OTC, deriving higher-titer genealogies that led to the contemporary industrial strains that are now responsible for the production of >10^8^ kg of OTC annually. Stimulated by commercial interest over the years, a sophisticated genetics and molecular biology has been developed for *S. rimosus* (5). This announcement reports the derivation of data to enable the generation of a scaffold genome sequence for this important industrial species.

Genome sequencing of the *S. rimosus* type strain ATCC 10970 was carried out using a whole-genome shotgun sequencing approach performed on a Roche 454 GS Junior apparatus. Using three single-ended runs, we obtained 440,253 reads. The reads were assembled using Genome Sequencer *de novo* Assembler (version 2.7, Roche), which led to a final assembly of 453 contigs of >500 bp. The total size of the assembly was 9.5 Mbp, with a mean contig size of 21 kbp (average, 17× coverage) and a G+C content of 71.88%. Automatic functional annotation results were obtained using the NCBI Prokaryotic Genome Annotation Pipeline (http://www.ncbi.nlm.nih.gov/genomes/static/Pipeline.html).

The draft genome of *S. rimosus* is estimated to have a total of 8,416 protein-coding genes, along with 66 tRNAs. The overall genome size is consistent with the physical map of *S. rimosus*, derived by pulsed-field gel electrophoresis (6). It is also within the range of other published streptomycete genome sequences. Three gene clusters associated with the production of previously reported secondary metabolites [OTC (7), rimocidin (8), and desferrioxamine C (9)] are readily identified within the sequence, in addition to around 45 other putative secondary metabolite biosynthetic clusters whose functions remain to be elucidated (10). The strain is lysogenic for phages RP2 and RP3, with the DNA sequences corresponding to the restriction maps published previously (11). The genome sequence is also likely to include at least one giant (387 kb) linear plasmid known to be present in *S. rimosus* (12).

Sequencing of *S. rimosus* ATCC 10970 provides a scaffold from which to investigate and understand how mutation results in higher titers of OTC.

### Nucleotide sequence accession numbers.

The *S. rimosus* ATCC 10970 Whole Genome Shotgun project has been deposited at DDBJ/EMBL/GenBank under the accession no. ANSJ00000000. The version described in this paper is the first version, ANSJ01000000.
